# Supplementary material for: Successful treatment of diplopia using prism correction combined with vision therapy/orthoptics improves health-related quality of life
Source: PeerJ. 2024 May 9;12:e17315. doi: 10.7717/peerj.17315 (PMC11088820; doi:10.7717/peerj.17315)
Supplement: Supplemental Information 1 — Different variables at baseline visit. Abbreviations: BCVA = Best Corrected Visual Acuity in decimal units; RX = refraction in spheric equivalent diopters; ADD = progressive addition lenses in spheric diopters; RPST = random-dot preschool stereoacuity test in seconds of arc; Worth = Worth Four Dot test with three possible responses (fusion: 4 dots; diplopia: 5 dots; and suppression: 2 or 3 dots). [file peerj-12-17315-s001.docx]

Annex 1. Different variables at baseline visit. Abbreviations: BCVA = Best Corrected Visual Acuity in decimal units; RX = refraction in spheric equivalent diopters; ADD = progressive addition lenses in spheric diopters; RPST = random-dot preschool stereoacuity test in seconds of arc; Worth = Worth Four Dot test with three possible responses (fusion: 4 dots; diplopia: 5 dots; and suppression: 2 or 3 dots).

| N | Age  (years) | Sex | Diplopia  duration  (months) | RX  Right eye | RX  Left eye | ADD | VA  Right eye | VA  Left eye | Worth | RPST |
| --- | --- | --- | --- | --- | --- | --- | --- | --- | --- | --- |
| 1 | 55 | M | 24 | 0.75 | -1.50 | 0.00 | 1 | 0,45 | 5 | 1300 |
| 2 | 78 | M | 48 | 0.5 | 1 | 2.50 | 1 | 0.9 | 5 | 1300 |
| 3 | 82 | M | 36 | 0.75 | -0.50 | 2.75 | 0.8 | 1 | 5 | 100 |
| 4 | 62 | F | 120 | -1.25 | 2.25 | 2.50 | 1 | 1 | 5 | 100 |
| 5 | 41 | M | 24 | -3 | -3 | 0,00 | 1 | 1 | 5 | 40 |
| 6 | 26 | F | 12 | 2.5 | -3.5 | 0,00 | 1 | 1 | 5 | 800 |
| 7 | 85 | F | 60 | -0.75 | 0 | 2.50 | 1 | 1 | 5 | 100 |
| 8 | 58 | F | 4 | 0 | 0.25 | 2.00 | 1 | 1 | 5 | 100 |
| 9 | 46 | M | 12 | -2.00 | -1.75 | 0,00 | 1 | 1 | 5 | 100 |
| 10 | 79 | F | 36 | 0.5 | 1.5 | 2.50 | 1 | 0.96 | 5 | 100 |
| 11 | 64 | F | 4 | -1.25 | -2.25 | 2.00 | 1 | 1 | 5 | 1300 |
| 12 | 41 | F | 48 | -16.00 | 16.00 | 1,50 | 0.94 | 0.84 | 5 | 1300 |
| 13 | 76 | M | 60 | 2.25 | 2.25 | 2.50 | 0.8 | 0.7 | 5 | 1300 |
| 14 | 59 | F | 12 | 0 | 0 | 2.50 | 1 | 1 | 5 | 100 |
| 15 | 35 | M | 4 | 0 | 0 | 0 | 1 | 1 | 5 | 1300 |
| 16 | 35 | M | 36 | 0 | 0 | 0 | 1 | 1 | 4 | 1300 |
| 17 | 74 | M | 6 | -1 | -3 | 2.50 | 0.7 | 1 | 5 | 1300 |
| 18 | 74 | M | 12 | -0.25 | 1 | 2.50 | 0.68 | 0.42 | 5 | 1300 |
| 19 | 80 | M | 12 | -0.50 | -0.75 | 0 | 1 | 1 | 5 | 100 |
| 20 | 43 | M | 36 | 0 | 0.25 | 0 | 1 | 1 | 5 | 100 |
| 21 | 69 | F | 24 | 2 | 3.25 | 2.50 | 1 | 0.9 | 5 | 1300 |
| 22 | 86 | M | 24 | -0.5 | -0.25 | 2.50 | 0.7 | 0.84 | 5 | 1300 |
| 23 | 79 | M | 5 | 4.75 | -1.75 | 3.00 | 0.44 | 0.9 | 5 | 100 |
| 24 | 31 | M | 12 | 0 | 0 | 0 | 1 | 1 | 5 | 1300 |
| 25 | 65 | F | 8 | -0.50 | -0.25 | 2.50 | 1 | 1 | 5 | 60 |
| 26 | 57 | M | 16 | 1 | 1.5 | 2.25 | 0.9 | 0.9 | 5 | 1300 |
| 27 | 71 | F | 20 | 4.25 | 3.5 | 2.50 | 0.84 | 0.84 | 4 | 1300 |
| 28 | 75 | M | 6 | 1.5 | 0.75 | 2.50 | 1 | 1 | 5 | 1300 |
| 29 | 54 | M | 36 | -5.75 | -6 | 2.00 | 0.9 | 0.9 | 5 | 1300 |
| 30 | 64 | F | 6 | 0 | 0.25 | 2.50 | 1 | 0.96 | 5 | 1300 |
| 31 | 31 | M | 36 | 1 | 1 | 0 | 1 | 1 | 5 | 1300 |
| 32 | 63 | F | 4 | 1.5 | 1.25 | 1.75 | 0.9 | 1 | 5 | 1300 |
| 33 | 78 | M | 60 | 1.5 | 1 | 2.50 | 1 | 1 | 5 | 100 |
| 34 | 69 | M | 3 | 1 | 0.25 | 2.50 | 1 | 1 | 5 | 1300 |
| 35 | 71 | F | 60 | 1.25 | 2.5 | 3.00 | 0.4 | 0.8 | 5 | 1300 |
| 36 | 66 | M | 24 | -3 | -2.75 | 2.50 | 1 | 1 | 5 | 1300 |
| 37 | 80 | M | 36 | -1 | -3.25 | 3.00 | 0.92 | 0.7 | 5 | 1300 |
| 38 | 64 | F | 14 | -22 | -22 | 2.50 | 1 | 0.9 | 5 | 1300 |
| 39 | 69 | M | 20 | 0.75 | -1.5 | 3.00 | 0.98 | 0.96 | 5 | 1300 |
| 40 | 60 | F | 60 | 2.25 | 1.5 | 2.25 | 1 | 1 | 5 | 1300 |
| 41 | 80 | F | 42 | 0.5 | 0.5 | 3,00 | 1 | 1 | 5 | 200 |
| 42 | 59 | M | 60 | -1,75 | -1,75 | 2,00 | 1 | 1 | 5 | 1300 |
| 43 | 54 | F | 24 | 1,125 | 1 | 2,00 | 1 | 1 | 5 | 1300 |
| 44 | 73 | F | 24 | 0 | 0 | 3 | 1 | 1 | 5 | 100 |
| 45 | 74 | M | 48 | -0,75 | 1 | 2,5 | 0,62 | 0,4 | 5 | 1300 |
| 46 | 34 | M | 4 | -4,5 | 0 | -4,5 | 1 | 1 | 5 | 1300 |
| 47 | 64 | F | 48 | -4 | -0,5 | 3 | 1 | 1 | 5 | 1300 |
| 48 | 65 | F | 24 | -0,5 | 0 | 2,5 | 0,48 | 1 | 5 | 1300 |
