# Supplementary material for: Successful treatment of diplopia using prism correction combined with vision therapy/orthoptics improves health-related quality of life
Source: PeerJ. 2024 May 9;12:e17315. doi: 10.7717/peerj.17315 (PMC11088820; doi:10.7717/peerj.17315)
Supplement: Supplemental Information 2 — Annex 2. Different variables at baseline visit. Abbreviations: HD = Horizontal deviation; ET = endotropia; XT = exotropia deviation; D = prismatic diopters; Hyper = hypertropia; Hypo = hypotropia; T = Torsional Deviation; PEO = progressive external ophthalmoplegia. [file peerj-12-17315-s002.docx]

Annex 2. Different variables at baseline visit. Abbreviations: HD = Horizontal deviation; ET = endotropia; XT = exotropia deviation; D = prismatic diopters; Hyper = hypertropia; Hypo = hypotropia; T = Torsional Deviation; PEO = progressive external ophthalmoplegia.

| N | Eye | HD Far | | VD Far | | HD Near | | VD Near | | T Deviation | | Diagnosis | Previous Treatment |
| --- | --- | --- | --- | --- | --- | --- | --- | --- | --- | --- | --- | --- | --- |
| 1 | Left | ET | 4 D | HYPER | 8 D | XT | 6 D | HYPER | 10 D | Excyclotorsion | 5° | 4th cranial nerve paresis | Strabismus surgery and botulinum toxin |
| 2 | Right | ET | 10 D | HYPER | 6 D | XT | 10 D | HYPER | 4 D | Excyclotorsion | 10° | 4th cranial nerve paresis | Botulinum toxin |
| 3 | Right | ET | 4 D | 0 | 0 | 0 | 0 | 0 | 0 | 0 | 0 | 6th cranial nerve paresis |  |
| 4 | Right | ET | 4 D | 0 | 0 | 0 | 0 | 0 | 0 | 0 | 0 | 6th cranial nerve paresis | Botulinum toxin |
| 5 | Right | 0 | 0 | HYPER | 2 D | 0 | 0 | 0 | 0 | 0 | 0 | Inferior oblique overaction |  |
| 6 | Right | XT | 15 D | 0 | 0 | XT | 25 D | 0 | 0 | 0 | 0 | Decompensation of strabismus | Strabismus surgery and Botulinum toxin |
| 7 | Right | ET | 10 D | 0 | 0 | 0 | 0 | 0 | 0 | 0 | 0 | 6th cranial nerve paresis | Botulinum toxin |
| 8 | Left | ET | 5 D | 0 | 0 | 0 | 0 | 0 | 0 | 0 | 0 | 6th cranial nerve paresis |  |
| 9 | Right | ET | 12 D | 0 | 0 | 0 | 0 | 0 | 0 | 0 | 0 | Restrictive strabismus | Graves ophthalmopathy surgery |
| 10 | Left | ET | 7 D | 0 | 0 | 0 | 0 | 0 | 0 | 0 | 0 | 6th cranial nerve paresis |  |
| 11 | Left | 0 | 0 | HYPER | 20 D | 0 | 0 | HYPER | 20 D | 0 | 0 | Inferior rectus underaction | Scleral buckling in retinal detachment |
| 12 | Left | ET | 30 D | HYPER | 4 D | ET | 30 D | HYPER | 4 D | 0 | 0 | Myopic restrictive myopathy | Strabismus surgery |
| 13 | Right | 0 | 0 | HYPER | 4 D | 0 | 0 | 2 Δ | 2 D | Excyclotorsion | 5° | 4th cranial nerve paresis | Ictus |
| 14 | Right | ET | 6 D | 0 | 0 | 0 | 0 | 0 | 0 | 0 | 0 | 6th cranial nerve paresis |  |
| 15 | Right | ET | 4 D | HYPER | 2 D | ET | 4 D | HYPER | 6 D | 0 | 0 | Inferior rectus underaction | Strabismus surgery |
| 16 | Right | 0 | 0 | 0 | 0 | XT | 25 D | 0 | 0 | 0 | 0 | PEO and convergence insufficiency |  |
| 17 | Right | ET | 12 D | 0 | 0 | ET | 6 D | 0 | 0 | 0 | 0 | 6th cranial nerve paresis | Botulinum toxin |
| 18 | Left | ET | 8 D | HYPER | 4 D | ET | 8 D | HYPER | 6 | 0 | 0 | 4th cranial nerve paresis |  |
| 19 | Left | ET | 4 D | 0 | 0 | 0 | 0 | 0 | 0 | 0 | 0 | 6th cranial nerve paresis |  |
| 20 | Right | ET | 4 D | 0 | 0 | 0 | 0 | 0 | 0 | 0 | 0 | 6th cranial nerve paresis | Botulinum toxin |
| 21 | Left | 0 | 0 | HYPER | 6 D | 0 | 0 | HYPER | 6 D | 0 | 0 | 4th cranial nerve paresis |  |
| 22 | Left | XT | 4 D | 0 | 0 | XT | 10 D | 0 | 0 | 0 | 0 | Convergence insufficiency |  |
| 23 | Right | ET | 6 D | 0 | 0 | 0 | 0 | 0 | 0 | 0 | 0 | 6th cranial nerve paresis |  |
| 24 | Left | ET | 16 D | HYPER | 6 D | ET | 16 D | HYPER | 6 D | 0 | 0 | Decompensation of strabismus | Strabismus surgery |
| 25 | Left | ET | 4 D | 0 | 0 | 0 | 0 | 0 | 0 | 0 | 0 | 6th cranial nerve paresis |  |
| 26 | Left | ET | 20 D | HYPER | 2 D | ET | 16 D | HYPER | 2 D | 0 | 0 | 6th cranial nerve paresis | Strabismus surgery and botulinum toxin |
| 27 | Left | 0 | 0 | 0 | 0 | XT | 12 D | 0 | 0 | 0 | 0 | Convergence insufficiency |  |
| 28 | Left | 0 | 0 | HYPER | 4 D | 0 | 0 | HYPER | 4 D | 0 | 0 | 4th cranial nerve paresis |  |
| 29 | Left | ET | 12 D | 0 | 0 | ET | 4 D | 0 | 0 | 0 | 0 | 6th cranial nerve paresis |  |
| 30 | Right | 0 | 0 | HYPER | 2 D | 0 | 0 | HYPER | 4 D | Excyclotorsion | 5° | 4th cranial nerve paresis | Botulinum toxin |
| 31 | Left | 0 | 0 | HYPER | 6 D | 0 | 0 | HYPER | 6 D | Excyclotorsion | 8° | 4th cranial nerve paresis |  |
| 32 | Right | 0 | 0 | HYPER | 16 D | 0 | 0 | HYPER | 16 D | 0 | 0 | Inferior rectus underaction |  |
| 33 | Left | 0 | 0 | HYPER | 4 D | 0 | 0 | HYPER | 4 D | Excyclotorsion | 4° | 4th cranial nerve paresis |  |
| 34 | Right | ET | 20 D | HYPER | 8 D | ET | 10 D | HYPER | 8 D | Excyclotorsion | 4° | 6th cranial nerve paresis | Botulinum toxin |
| 35 | Right | 0 | 0 | HYPO | 10 D | 0 | 0 | HYPO | 10 D | 0 | 0 | Inferior rectus overaction | Botulinum toxin |
| 36 | Left | 0 | 0 | HYPER | 4 D | 0 | 0 | HYPER | 4 D | 0 | 0 | 4th cranial nerve paresis |  |
| 37 | Left | 0 | 0 | HYPER | 12 D | 0 | 0 | HYPER | 12 D | Excyclotorsion | 4° | 6th cranial nerve paresis |  |
| 38 | Left | ET | 4 D | HYPO | 2 D | ET | 4 D | HYPO | 3 D | 0 | 0 | Myopic restrictive myopathy | Strabismus surgery |
| 39 | Left | 0 | 0 | HYPER | 8 D | 0 | 0 | HYPER | 8 D | Excyclotorsion | 2° | 4th cranial nerve paresis |  |
| 40 | Right | 0 | 0 | HYPER | 8 D | 0 | 0 | HYPER | 8 D | Excyclotorsion | 6° | 4th cranial nerve paresis |  |
| 41 | Right | ET | 4 D | 0 | 0 | 0 | 0 | 0 | 0 | 0 | 0 | 6th cranial nerve paresis |  |
| 42 | Right | XT | 12 D | HYPO | 10 D | XT | 10 D | HYPO | 10 D | Excyclotorsion | 10° | Inferior rectus overaction | Botulinum toxin |
| 43 | Left | ET | 6 D | 0 | 0 | ET | 4 | 0 | 0 | 0 | 0 | 6th cranial nerve paresis |  |
| 44 | Left | ET | 6 D | 0 | 0 | 0 | 0 | 0 | 0 | 0 | 0 | 6th cranial nerve paresis |  |
| 45 | Left | ET | 6 D | HYPER | 4 D | ET | 6 | HYPER | 2 D | Excyclotorsion | 4° | 4th cranial nerve paresis |  |
| 46 | Left | 0 | 0 | HYPER | 8 D | 0 | 0 | HYPER | 8 D | Excyclotorsion | 2° | 4th cranial nerve paresis |  |
| 47 | Left | 0 | 0 | HYPER | 4 D | 0 | 0 | HYPER | 4 D | Excyclotorsion | 5° | 4th cranial nerve paresis |  |
| 48 | Right | 0 | 0 | HYPER | 6 D | 0 | 0 | HYPER | 8 D | 0 | 0 | Inferior rectus underaction |  |
